# Supplementary material for: Prohibiting Babel—A call for professional remote interpreting services in pre-operation anaesthesia information
Source: PLoS One. 2025 Jan 17;20(1):e0299751. doi: 10.1371/journal.pone.0299751 (PMC11741601; doi:10.1371/journal.pone.0299751)
Supplement: S1 File — (DOCX) [file pone.0299751.s001.docx]

**Supporting Information**

**Sup A: List of lay interpreters.**

The lay interpreters consisted of: 4x spouse; 17x sibling, 1x grandchild, 1x parent, 1x cousin, 3x friend, 1x work colleague, 1x physician’s assistant, 1x not known.
